# Paraquat Toxicity Leading to Acute Kidney Injury: A Case Report

**DOI:** 10.31729/jnma.8560

**Published:** 2024-05-31

**Authors:** Newton Ashish Shah, Manish Yadav, Rupesh Shah, Bibek Rajbhandari

**Affiliations:** 1 Maharajgunj Medical Campus, Kathamandu, Nepal; 2 Universal College of Medical Sciences, Bhairahwa, Nepal; 3 Tribhuvan University Teaching Hospital, Kathmandu, Nepal

**Keywords:** *acute renal injury*, *paraquat*, *toxicology*

## Abstract

Paraquat poisoning poses a significant and emerging public health challenge in developing countries. The distribution and usage of Paraquat, a potent herbicide, remain unrestricted in many regions despite its high fatality rate and absence of a specific antidote. Paraquat mostly involves the lungs but can also involve the kidneys and liver. Diagnostic challenges and a lack of available samples at presentation contribute to underreporting and limited awareness among healthcare providers, making paraquat poisoning a neglected toxicological emergency. Herein, we present a case of a 40-year-old male who presented to the emergency department on the fourth day after ingesting paraquat in a suicidal attempt. Upon presentation, he had erosion on the tongue and posterior pharyngeal wall, along with deranged renal function tests and elevated serum creatinine levels. The patient developed acute kidney injury, with serum creatinine levels rapidly rising from normal to 3.85 mg/dl, accompanied by a decrease in daily urine output. He was managed conservatively, and his hospital stay was uneventful.

## INTRODUCTION

Poisoning is the second most common method of suicide in Nepal, with 90% of cases due to pesticides.^1^ The distribution and usage of Paraquat, a potent herbicide, remain unrestricted in many regions, despite its high fatality rate and the absence of a specific antidote, posing a significant and emerging public health challenge in developing countries. Paraquat's toxicity mostly involves the lungs but can also affect other organs, including the kidneys.^2^ Renal involvement are seen in 20-70% of cases depending on ingested dose and time of presentation.^3^ We present a case of 40-year-old who ingested paraquat and presented with delayed-onset AKI.

## CASE REPORT

**Figure 1:**
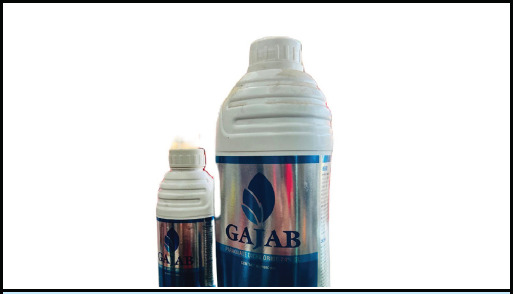
Paraquat container brought by patient.

A 40-year-old previously healthy male presented to our Emergency Room (ER) with a reported history of intentional paraquat ingestion approximately 4 days prior. The patient stated ingesting an estimated 15 ml of Paraquat dichloride 24% soluble liquid (SL) with suicidal intent with no prior attempts (Figure 1). He had initially sought medical care at a nearby facility and had undergone conservative management. Symptoms reported by the patient included episodes of vomiting, abdominal pain, and throat discomfort. Notably, there were no complaints of fever, loss of consciousness, headache, or jaundice. Additionally, the patient noted passing black tarry stools. The patient disclosed a chronic history of alcohol consumption, averaging approximately 70 grams daily over 5 years.

**Figure 2:**
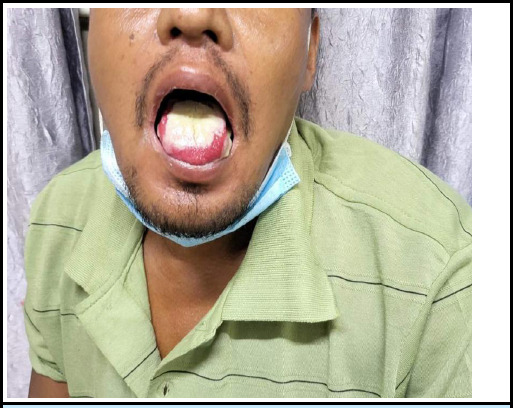
Sloughing of tongue mucosa showing "Paraquat Tongue".

Upon physical examination, the patient appeared to be in fair overall condition. Notable findings included erosion observed on the tongue and posterior pharyngeal wall (Figure 2). However, there were no signs of pallor, jaundice, or cyanosis. His blood pressure was 120/90 mm of Hg, with a respiratory rate of 18 breaths/minute and, pulse rate of 88 beats/minute. Oxygen saturation was recorded at 94% on room air. Systemic examinations revealed no other remarkable findings.

Initial investigations included an ultrasound scan, which showed unremarkable results. Electrocardiography demonstrated normal sinus rhythm. Laboratory findings revealed abnormal stool coloration consistent with the passage of black tarry stools, indicative of upper gastrointestinal bleeding. Blood investigations were done which showed following values (Table 1). Further laboratory tests were conducted to assess renal function, hepatic enzymes, and electrolyte levels. Urea, and creatinine were persistently higher (Table 2) with all other blood tests within normal limits.

**Table 1:** Laboratory investigations.

Parameters	Value	Normal Range
Hemoglobulin	12.9	12-18 mg/dL
Total Leucocyte count	7100	4000-11000/cmm
Platelets	331000	150000-450000/cumm
PT/INR	14.5/1.08	
Glucose Random	6.4	3.8-7.8 mmol/L
Direct Bilirubin	5	<4
SGPT	24	0-50 U/L
SGOT	27	0-50 U/L
Alkaline Phosphatase	72	30-120 U/L
Total Protein	67	66-83 gm/L

**Table 2:** Subsequent urea and creatinine values.

Days (from Ingestion)	Urea (2.8-7.2 mmol/L)	Creatinine (74-110 uMol/L)
Day 5	9.20	340
Day 6	9.8	353
Day 9	12.8	310

**Figure 3:**
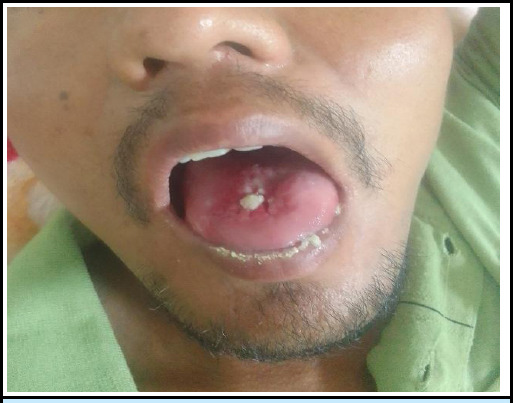
Resolution phase of paraquat poisoning showing healing mucosa of tongue.

Given the patient's presentation and history of paraquat ingestion, immediate measures were taken to manage symptoms and mitigate potential systemic effects. Fluid resuscitation was initiated Normal saline bolus then at 100 ml/hr and one pint of Dextrose Normal Saline (DNS) to address dehydration and support meta bolicprocesses complicating AKI. Intravenous dexamethasone was initiated along with IV n-acetylcysteine (NAC). Injectable tramadol was incorporated as needed for pain management for abdominal pain and throat pain. Plans were made for hemodialysis if fluid resuscitation proved ineffective, but ultimately, the patient responded well to fluids. Additionally, measures were taken to address gastrointestinal symptoms, including administration of proton pump inhibitor (pantoprazole 40 mg) and antiemetics (ondansetron 5 mg). Oral ulcers were treated with a gel containing a combination of Lidocaine (2% w/w), Chlorhexidine Gluconate (1% w/w), and Metronidazole (1% w/w). The sloughed off mucose healed gradually (Figure 3). Close monitoring of the patient's clinical status and response to treatment was instituted to guide ongoing management decisions. The patient exhibited a positive recovery trajectory and was referred to a psychiatrist for counseling. Subsequent follow-up appointments showed a normal course of improvement.

## DISCUSSION

Paraquat (N, N'-dimethyl-4,4'-bipyridinium dichloride), is an effective herbicide that is a brown solution with a strong odor. It accounts for 13% of all deaths due to poisoning.^4^ Currently no specific effective antidote exists for paraquat poisoning, underscoring the critical role of early diagnosis, decontamination, and aggressive symptomatic management.^5^ For this reason, the use has been restricted in many parts of the world. However, cases are largely reported from areas where agriculture is practiced by a majority of the population. This may be due to the easy availability and accessibility of paraquat in those regions. Ingestion of even a small amount (i.e., 10 to 20 ml of 20% solution) is considered lethal. A plasma concentration of >1.6 pg/ml, 12 hours after administration, has been determined to be always lethal. In the context of our presented case, paraquat was ingested with the intent of suicide causing Acute Kidney injury (AKI). The incidence of AKI in such cases, existing literature suggests a variable rate ranging from 20% to 70%, depending on factors such as the dose of paraquat ingested, time to presentation, and adequacy of medical management.^3^

Poisoning can be either through the topical route or by ingestion. Clinical manifestations that follow paraquat poisoning depend upon the route and amount of ingestion. Paraquat Poisoning presents itself in three stages. At first, symptoms include headaches, burning sensations at the site of contact, stomach issues, and potentially fluid buildup in the lungs. The next phase involves harmful effects on the liver and kidneys, and in uncommon instances, damage to the heart. The onset of pulmonary fibrosis marks the beginning of the third stage.^2^ Ingestion of large amounts can lead to multiple organ failure causing pulmonary edema, cardiac failure, renal failure or hepatic failure, and convulsions due to CNS involvement.^2^

The principal etiology of its toxicity resides in its capacity for redox cycling, engendering heightened Reactive Oxygen Species (ROS) production. This propensity amplifies oxidative stress, culminating in lipid peroxidation and, ultimately, impeding cellular membranes and inducing apoptotic pathways which can cause tubule cell injury in both ischemic and toxic acute tubular necrosis.^5^ Similarly, the body's efforts to eliminate paraquat can lead to impaired renal function. Within our presented case, Renal Function Tests were deranged indicating acute kidney injury.

Diagnosis of paraquat poisoning is commonly predicated on circumstantial evidence and clinical manifestations. Noteworthy diagnostic indicators include the patient's history of paraquat exposure, the presence of empty pesticide containers, suggestive residues, distinctive odors, or anomalous coloring. Urinary Dithionite Test can be expedited at the patient's bedside. Under alkaline conditions, sodium dithionite precipitates paraquat reduction, generating a discernible blue radical.^5^ The patient's presentation with erosion on the tongue and posterior pharyngeal wall, along with gastrointestinal symptoms, initially obscured the diagnosis of paraquat poisoning. These atypical symptoms deviated from the classic respiratory or hepatic manifestations typically associated with paraquat ingestion, posing a diagnostic challenge. In resource-limited settings, availability and accessibility of diagnostic tests for paraquat poisoning, such as urinary dithionite testing or plasma paraquat levels, may be limited. This can impede timely confirmation of the diagnosis and initiation of appropriate treatment. In our case, patient party had retrieved the poisoning bottle which aided to our diagnosis. (Figure 1)

Presently, there are no established protocols for treating paraquat toxicity owing to the absence of an antidote and the infrequency of poisonings. The case fatality rate can be as high as around 50% to 90%.^6^ Symptomatic treatment remains the mainstay treatment, focusing mainly on decontamination and stabilization of the patient. Gastric decontamination is beneficial in those patients who present within 1-2 hours of ingestion. Activated charcoals can be used. However, lavage is contraindicated due to its corrosive nature. Pulse therapy with cyclophosphamide and methylprednisolone has shown effective in preventing organ failure and reducing mortality by 25%. Pulse therapy, along with antioxidants such as N-acetyl-cysteine, and vitamin C has shown efficacy in preventing ongoing inflammation with varied outcomes .^7^ Acute hepatitis is a significant predictor of AKI after paraquat intoxication which warrants careful observation of Liver function Test. In our patient, LFT were within normal limit.^8^

Patients exhibiting the onset of acute renal failure warrant prompt intervention in the form of hemodialysis until the restoration of normal renal function is achieved. Fluid resuscitation is equally important to correct electrolytes. With adequate treatment, creatinine levels take around 3 weeks to return to normal range. According to Kim et al, initial serum Cr level greater than 1.2 mg/dL can be a significant predictor of mortality.^9^ Baseline serum uric acid level can be a good clinical marker for patients at risk of mortality and AKI after acute PQ intoxication. Studies show hyperuricemia patients have increased the risk of mortality and kidney failure to 3.7- and 3.3- fold.^10^

The use of paraquat as a method for suicide poses a significant public health challenge owing to its potent toxicity and absence of effective antidotes. Despite regulatory efforts aimed at restricting access to paraquat and raising awareness of its hazards, instances of paraquat-related suicides persist, underscoring the pressing necessity for comprehensive prevention strategies. Such strategies could encompass stricter regulations governing paraquat availability, bolstered mental health support services, and community education initiatives aimed at dissuading its utilization for self-harm. Furthermore, research efforts to develop alternative herbicides characterized by lower toxicity profiles could play a pivotal role in mitigating the incidence of paraquat-related suicides and safeguarding public health.^5^ However, despite advancements, the challenges persist, as demonstrated by the complexities of managing paraquat poisoning, including multiorgan failure and the lack of specific antidotes, highlighting the critical need for ongoing research to enhance diagnostic tools and therapeutic strategies.
